# Diabetic Pregnancy and Maternal High-Fat Diet Impair Mitochondrial Dynamism in the Developing Fetal Rat Heart by Sex-Specific Mechanisms

**DOI:** 10.3390/ijms20123090

**Published:** 2019-06-25

**Authors:** Tricia D. Larsen, Kyle H. Sabey, Alexis J. Knutson, Tyler C. T. Gandy, Eli J. Louwagie, Lothar Lauterboeck, Kennedy S. Mdaki, Michelle L. Baack

**Affiliations:** 1 Environmental Influences on Health and Disease Group, Sanford Research, 2301 E 60th Street North, Sioux Falls, SD 57104, USA; tricia.larsen@sanfordhealth.org (T.D.L.); ajknutson15@ole.augie.edu (A.J.K.); tyler.gandy@sanfordhealth.org (T.C.T.G.); eli.louwagie@sanfordhealth.org (E.J.L.); llaute@lsuhsc.edu (L.L.); mdaki@uthscsa.edu (K.S.M.); 2 Sanford School of Medicine of the University of South Dakota, Sioux Falls, SD 57117, USA; kyle.sabey@sanfordhealth.org; 3 Boekelheide Neonatal Intensive Care Unit, Sanford Children’s Hospital, Sioux Falls, SD 57117, USA; 4 Barshop Institute for Longevity and Aging Studies, UT Health, San Antonio, TX 78245, USA

**Keywords:** maternal diabetes, maternal high-fat diet, cardiovascular disease, mitochondrial dynamism, sex-specific mechanisms of the developmental origins of health and disease (DOHaD)

## Abstract

Infants born to diabetic or obese mothers are at greater risk of heart disease at birth and throughout life, but prevention is hindered because underlying mechanisms remain poorly understood. Using a rat model, we showed that prenatal exposure to maternal diabetes and a high-fat diet caused diastolic and systolic dysfunction, myocardial lipid accumulation, decreased respiratory capacity, and oxidative stress in newborn offspring hearts. This study aimed to determine whether mitochondrial dynamism played a role. Using confocal live-cell imaging, we examined mitochondrial dynamics in neonatal rat cardiomyocytes (NRCM) from four prenatally exposed groups: controls, diabetes, high-fat diet, and combination exposed. Cardiac expression of dynamism-related genes and proteins were compared, and gender-specific differences were evaluated. Findings show that normal NRCM have highly dynamic mitochondria with a well-balanced number of fusion and fission events. Prenatal exposure to diabetes or a high-fat diet impaired dynamism resulting in shorter, wider mitochondria. Mechanisms of impaired dynamism were gender-specific and protein regulated. Females had higher expression of fusion proteins which may confer a cardioprotective effect. Prenatally exposed male hearts had post-translational modifications known to impair dynamism and influence mitophagy-mediated cell death. This study identifies mitochondrial fusion and fission proteins as targetable, pathogenic regulators of heart health in offspring exposed to excess circulating maternal fuels.

## 1. Introduction

Infants born to women with diabetic pregnancy (IDM), obesity, or both, are at greater risk of heart disease including cardiomyopathy at birth [1,2,3,4,5,6,7,8,9] and throughout life [10,11,12,13,14,15,16,17,18]. While cardiomyopathy is rare among infants born to non-diabetic mothers [19], up to one third of IDM have cardiac dysfunction at birth [5] and some develop severe or even fatal cardiomyopathy [20,21,22]. Those surviving improve but remain at higher risk of cardiovascular disease when they are adults [11,13,17,18]. While it is well-recognized that diabetic pregnancy increases the risk of heart disease in the developing baby, prevention and treatment are significantly hindered because the underlying mechanisms remain unknown.

**Figure 1:**
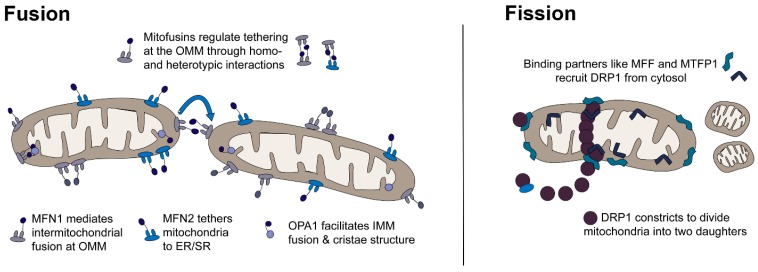
Mitochondria are highly dynamic, which allows adaptive energy production under changing metabolic conditions (starvation, feeding, exercise, rest, high and low oxygen supply). Mitochondria sense metabolic signals to trigger fusion (joining) and fission (division) which allows a change in their number, shape, and function through replication, culling, and recycling of mtDNA. Fusion (**left**) is regulated by homotypic and heterotypic interactions between dynamin related GTPases including mitofusin (MFN) 1 and 2 at the outer mitochondrial membrane (OMM) and optic atrophy (OPA1) at the inner mitochondrial membrane (IMM). Fission (**right**) is mediated by recruitment of dynamin related protein (DRP1) from the cytosol to binding proteins including OMM, mitochondrial fission factor (MFF), and IMM mitochondrial fission process 1 (MTFP1).

Mitochondrial dynamism is the intracellular movement of mitochondria for fusion and fission, and it plays an important role in both cardiac development and long-term heart health [23]. Dynamism influences mitochondrial number, morphology, function, and cell fate through important processes including mitochondrial replication (biogenesis), intracellular organelle communication, reticular arrangement (subsarcolemmal, myofibrillar, and perinuclear migration), cristae structure, respiratory complex assembly, mitochondrial DNA homeostasis, quality control (mitophagy or culling), and cell fate through apoptosis and necrosis [23,24,25,26,27,28,29,30,31,32,33]. As shown in Figure 1, dynamism is regulated by GTPase proteins located in the cytosol, outer mitochondrial membrane (OMM), and inner mitochondrial membrane (IMM).

Fusion (joining) is regulated by mitofusin 1 and 2 (MFN1, MFN2) and optic atrophy 1 (OPA1) [34]. MFN1 and MFN2 are transmembrane proteins bound to the OMM. They aid in mitochondria–mitochondria and mitochondria–organelle fusion by forming homotypic (MFN1–MFN1 or MFN2–MFN2) and heterotypic (MFN1–MFN2, MFN1–OPA1) complexes that link membranes [26,34]. Fusion is influenced by post-translational modifications including mitofusin ubiquitination which interferes with fusion and can result in unopposed fission [26,35]. MFN2 has additional non-dynamic roles in mitochondrial-endoplasmic/sarcoplasmic reticulum (ER/SR) communication, calcium flux, and cell survival through mitophagy induced apoptosis and necrosis pathways [26]. Fusion at the IMM is regulated by OPA1 which also regulates cristae structure and mitophagy induced apoptosis under the influence of oligomerization, splicing, and other post-translational modifications [23,26,28,36,37]. Fission (division) is primarily regulated by dynamin related protein 1 (DRP1), also known as dynamin-1-like protein (DNM1L), a cytosolic GTPase that is recruited to OMM binding partners including mitochondrial fission factor (MFF) [26,36]. Phosphorylation of DRP1 at Ser^637^ blocks recruitment and prevents fission [36]. Mitochondrial fission process 1 (MTFP1) and death associated protein 3 (DAP3) influence fission and mitophagy through DRP1 phosphorylation and recruitment [38,39,40]. It is not known how exposure to maternal diabetes or dyslipidemia may affect mitochondrial dynamism, thus cardiac development and disease risk.

Our lab and others have described a normal transition of cardiac metabolism from fetal to adult life [41,42]. Using a rat model, we also demonstrated that in utero exposure to maternal diabetes and a high-fat diet was associated with diastolic and systolic dysfunction, myocardial lipid accumulation, decreased glycolytic and respiratory capacity, oxidative stress, and mitochondrial fragmentation in newborn offspring hearts [43]. Our findings mimicked those described in models of adult diabetic cardiomyopathy (left ventricular dysfunction independent of hypertension or coronary artery disease) [44,45,46,47] which reportedly results from mitochondrial damage and impaired dynamism following exposure to long-standing diabetes and dyslipidemia [44,48,49,50,51]. This study used a well-described rat model [42,43,52,53] to answer the critical question of whether maternal diabetes, diet, or both, can impair mitochondrial dynamism to influence cardiac health. Findings describe gender-specific differences and identify mitochondrial fusion and fission proteins as targetable, pathogenic regulators of cardiomyopathy in the fetus exposed to excess circulating maternal fuels.

## 2. Results

### 2.1. Maternal Diabetes or High-Fat Diet Impairs Mitochondrial Dynamism in the Developing Offspring’s Heart

**Figure 2:**
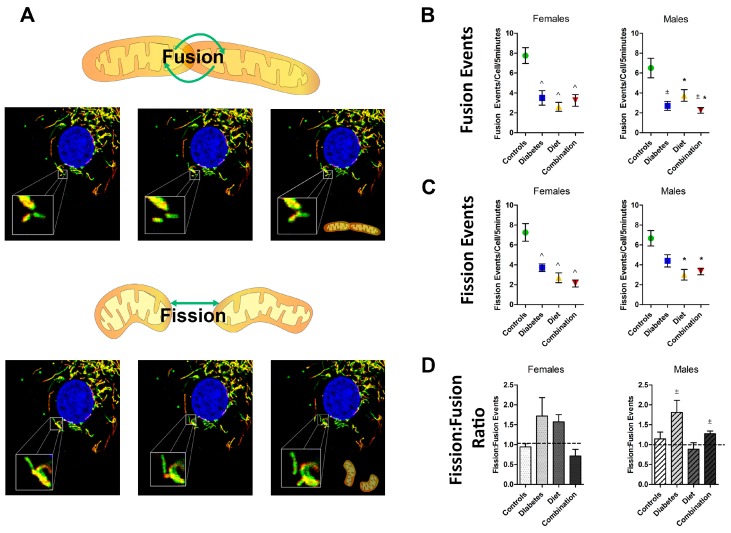
Maternal diabetes and a high-fat diet impaired mitochondrial dynamism in newborn rat cardiomyocytes (NRCM). Confocal live-cell imaging was used to count fusion and fission events of mitochondria in primary NRCM from controls and those prenatally exposed to maternal diabetes, a high-fat diet, or a combination of both. (**A**) Representative time lapsed images show mitochondrial fusion (joining of two separate mitochondria into one) in the top panels and fission (division of one mitochondrion into two) in the bottom panels. The average number of (**B**) fusion and (**C**) fission events/cell were counted in a 5 min recording. (**D**) A fission:fusion ratio was calculated to demonstrate imbalance and predict mitochondrial morphology. Group comparisons are shown for females (*n* = 4–7/group) and males (*n* = 4–6/group), respectively. Significant differences (*p* ≤ 0.05) are indicated for ^±^ diabetes and * diet by two-way ANOVA and ^ for interaction effect remaining by one-way ANOVA with comparison to controls by Dunnett’s post hoc analysis.

This study used live-cell imaging studies to objectively measure fusion and fission events in primary isolated neonatal rat cardiomyocytes (NRCM) from controls and offspring that were prenatally exposed to streptozocin-induced diabetes, maternal high-fat diet, or a combination of both. Representative videos are available as Video 1. The average number of fusion and fission events in each group is shown in Figure 2. Normal NRCM had highly dynamic mitochondria with approximately 1.4 paired fission and fusion events/cell/minute. Measuring fission and fusion as either the average number of events/cell/5 min video or as the events/100 µm^2^/5 min video to account for potential variance in cell size led to similar outcomes (Supplemental Figure S1). Dynamism was well balanced in normal NRCM with 7.00 ± 0.57 fusion events/cell and 6.99 ± 0.67 fission events/cell in 5 min of imaging leading to a balanced fission:fusion ratio of approximately 1 in controls. Maternal diabetes and/or a high-fat diet impaired mitochondrial dynamism in NRCM. On average, diabetes-exposed NRCM had 50% fewer fusion events and 30% fewer fission events with a pro-fission ratio of 1.8 (1.8 fission events for every 1 fusion event). Diet-exposed NRCM had 50% fewer fusion events and 44% fewer fission events with a similar trend in fission:fusion ratio (1.3). Combination exposed NRCM had a 50% decline in both fission and fusion, resulting in a balanced fission:fusion ratio of 0.99, but more mitochondria were adynamic and fragmented. While both fusion and fission were impaired, prenatal exposure to diabetes or a high-fat diet most often led to a pro-fission imbalance (Figure 2D).

### 2.2. Prenatal Exposures Alter Mitochondrial Morphology

**Figure 3:**
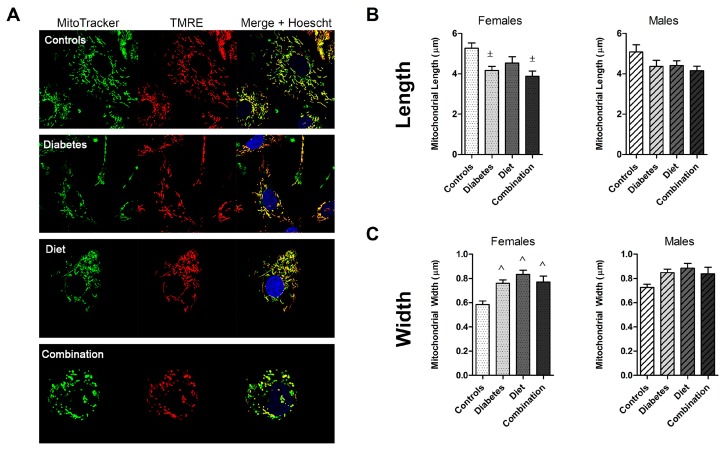
Prenatal exposure to diabetes and a high-fat diet alters cardiac mitochondrial morphology. (**A**) NRCM from control, diabetes, high-fat diet, and combination exposed offspring were stained with MitoTracker Green, tetramethylrhodamine ethyl ester (TMRE) (membrane potential), and Hoechst so that well-charged mitochondria appear yellow. (**B**) Mitochondrial length and (**C**) width from 20 mitochondria per sample (10 mitochondria/cell × 2 cells/sample and samples from individual litters) were measured and averaged. Group comparisons are shown for females (*n* = 4–7/group) and males (*n* = 4–6/group), respectively. Significant differences (*p* ≤ 0.05) are indicated with ^±^ for diabetes effect by two-way ANOVA and ^ for interaction effect with significance remaining by one-way ANOVA with comparison to controls by Dunnett’s post hoc analysis.

Differences in mitochondrial morphology are shown in Figure 3. As predicted by fission:fusion ratios, diabetes- and diet-exposed mitochondria were shorter and wider. Combination-exposed mitochondria had an equal decline in fission and fusion resulting in a balanced ratio, however, their mitochondria were also shorter and wider. By our observation, this was due to an overall higher number of poorly charged and adynamic (fragmented) mitochondria in that sub-group (Figure 3A).

### 2.3. Fetal Sex Influences Dynamic Events in Prenatally Exposed, but Not Normal Cardiomyocytes

To determine whether an offspring’s sex influenced dynamism and morphology, group comparisons were made separately for female and male offspring (Figure 2 and Figure 3). Gender-related differences were evaluated by *t*-test within each group (Table 1). Fetal sex did not influence the number of dynamic events or the balance of fission and fusion in normal NRCM. While normal males had slightly wider mitochondria, mitochondrial length was similar between sexes. Diabetes and diet impaired dynamism in both female and male NRCM. However, females had a more robust decline in mitochondrial fusion compared to males (3-fold fewer events compared to 1.5-fold fewer events). Since fission was equally impaired in diet-exposed NRCM, we found a gender-related divergence in mitochondrial balance. Female NRCM developed a pro-fission ratio (1.7), but males developed an anti-fission ratio (0.88). Despite equal decline in fission and fusion events, combination exposed female and male NRCM had significantly different fission:fusion ratios (Table 1), which may reflect differences in mitochondrial quality. Overall, our findings suggest that diabetes exposure impairs NRCM dynamism similarly in both sexes, but a maternal high-fat diet causes a sex-divergent response.

### 2.4. Expression of Genes Regulating Dynamism Do Not Explain Impaired Mitochondrial Dynamism

**Figure 4:**
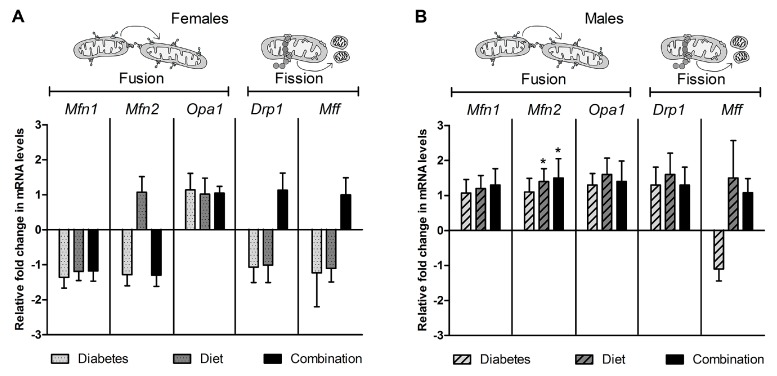
Cardiac expression of dynamism regulating genes cannot explain impaired dynamism in NRCM exposed to maternal diabetes, high-fat diet, or the combination of both. Whole heart mRNA was isolated from newborn offspring and relative expression of mitofusin 1 (*Mfn1*), mitofusin 2 (*Mfn2*), optic atrophy 1 (*Opa1*), dynamin related protein 1 (*Drp1*), and mitochondrial fission factor (*Mff*) was determined using qPCR; *B2m* was the reference gene. Bar graphs illustrate the average (mean ± SEM) fold change in expression compared to controls. Data are shown for (**A**) females (*n* = 8/group) and (**B**) males (*n* = 8–9/group), respectively. Significant (*p* < 0.05) group differences are shown for * diet effect by two-way ANOVA. There was no statistically significant difference when males and females were pooled.

To help delineate mechanisms of impaired dynamism, we compared whole heart mRNA expression of key genes regulating fusion (*Mfn1, Mfn2,* and *Opa1*) and fission (*Drp1* and *Mff*). As shown in Figure 4A, neither diabetes, nor high-fat diet exposure significantly influenced relative expression of dynamism regulatory genes.

To determine if an offspring’s sex influenced gene expression, we analyzed exposure related differences for females and males separately. In doing so, we found that diet-exposed male, but not female hearts had a higher *Mfn2* expression than controls (Figure 4B,C). While this does not explain impaired dynamism, it may partially explain why diet-exposed males have less of a decline in fusion than diet-exposed females. Gender-related differences within each group were also evaluated by *t*-test. There was no sex-specific differences in expression of cardiac dynamism related genes in the control, diabetes- or diet-exposed hearts. However, combination-exposed females had a higher expression of *Drp1* than combination-exposed males (Supplemental Table S3).

### 2.5. Cardiac Proteins Regulating Dynamism Are Influenced by Prenatal Exposure and Fetal Sex

**Figure 5:**
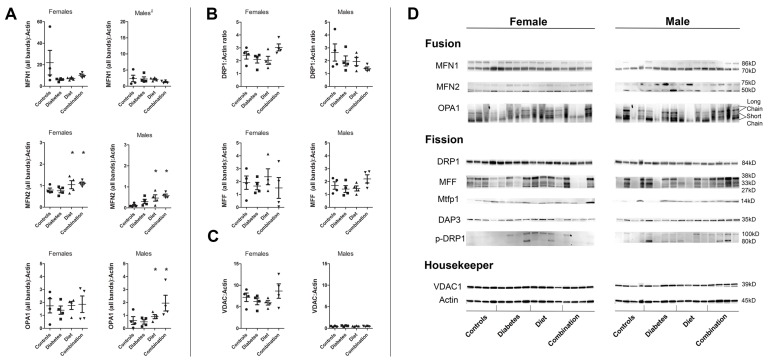
Maternal high-fat diet and diabetes influences cardiac expression of dynamism regulating proteins in a sex-specific manner. Whole heart protein was isolated and Western blot analysis was used to determine differences in relative expression of (**A**) fusion proteins MFN1, MFN2, OPA1 and (**B**) fission proteins, DRP1 and MFF. Single data points represent individual female or male offspring from different litters. Bars represent median and interquartile range. Data points show relative expression compared to the reference protein, β-actin. Regardless of exposure, females had significantly higher expression of all fusion proteins. (**C**) Voltage-dependent anion channel or porin (VDAC) was used as an additional mitochondrial housekeeper. VDAC:actin did not vary between exposure groups, however relative expression of VDAC was 16-fold higher in female compared to male hearts, which suggests a higher number of well-charged mitochondria per sample. (**D**) Immunoblots are shown. Significant group differences are annotated * for dietary effect by two-way ANOVA; *p* ≤ 0.05. *n* = 4 males and 4 females/group.

Next, we used Western blot to compare relative expression of cardiac proteins involved in fusion (MFN1, MFN2, and OPA1) and fission (DRP1, MFF, and MTFP1). When combining sexes for comparison, few differences were found. Diabetes- and diet-exposed offspring had a 50% lower MFN1 expression compared to controls; however, this trend did not reach statistical significance, likely due to inter-sample variance. Combined data also demonstrated that diet-exposed offspring had a significantly higher MFN2 expression. Western blot bands were variable between samples (Figure 5D). Since mitochondrial protein expression appeared variable between female and male offspring, male and female protein expression was analyzed separately to evaluate exposure related differences. High-fat diet-exposed hearts had higher MFN2 expression in males (44%); the trend was similar, but less robust in females (26%). Diet-exposed male, but not female, hearts also had higher OPA1 expression. These diet-related differences in protein expression may partially explain sex-divergent differences in fusion (females have a more robust decline in fusion than males).

When analyzing expression separately for females and males, we found that much of the protein expression variability was related to offspring sex. We used a *t*-test to determine gender-related differences within each group (Table 2). Results were surprising. Female hearts had a 2- to 9-fold higher expression of fusion proteins compared to male hearts; this difference spanned across nearly every group. Using voltage-dependent anion channel or porin (VDAC) as a mitochondrial reference protein, we found that female hearts had a higher expression of VDAC than male hearts across all groups. Indeed, normal females had a 16-fold higher expression of VDAC than normal males. Prenatal exposures did not change the relative expression of VDAC in offspring hearts which suggests this is specifically a gender-related difference. This sex-related difference remained when calculating VDAC:actin ratios (Figure 5C,D). To assure this was not related to changes in our housekeeper protein, we reanalyzed actin expression using several blots with samples from both sexes on the same gel. Consistently, actin expression was not different between females and males. We also compared mitochondrial copy number as previously described [43] and found no differences between control females and males. This data could suggest that females have a higher expression of fusion proteins and more well-charged (healthy) mitochondria compared to males.

### 2.6. Prenatal Exposure to High-Fat Diet Alters Dynamism in the Offspring’s Heart Following Sex-Specific Post-Translational Modifications of Fusion and Fission Proteins

**Figure 6:**
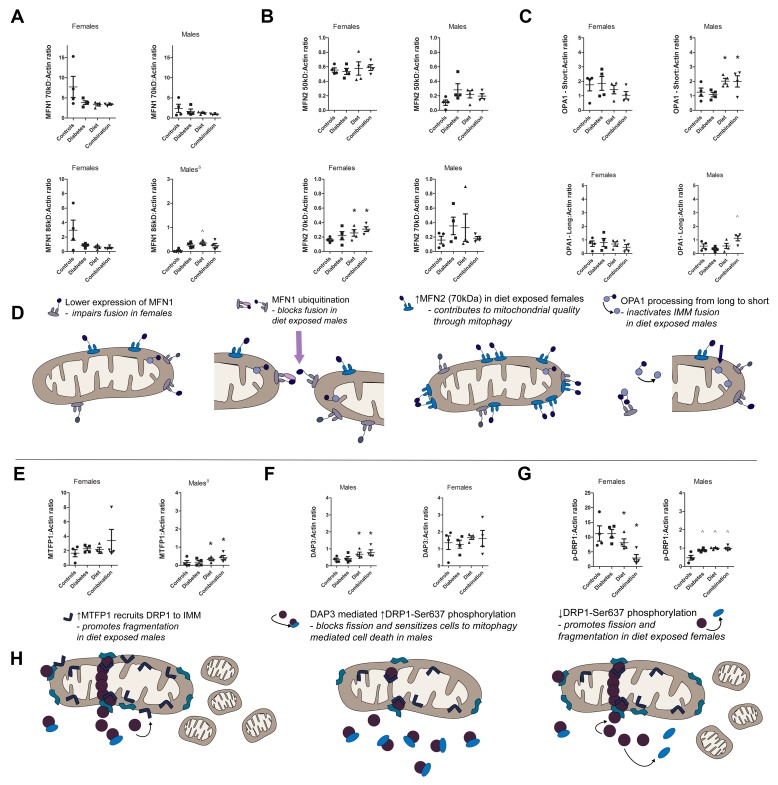
Post-translational modifications of dynamism regulatory proteins may explain altered dynamism in diabetes- and high-fat diet-exposed offspring hearts. Whole heart protein lysate from control, diabetes, high-fat diet, and combination exposed newborn rats was analyzed for known post-translational modifications known to affect (**A**–**D**) fusion and (**E**–**G**) fission. (**A**) MFN1 blots had two bands, the primary 70 kDa (active) band and an 86 kDa (inactive) diubiquitinated band (16 kDa higher) [35]. Diabetes- and diet-exposed females had a trend towards lower expression of both bands. Diet-exposed males had lower 70 kDa alongside higher 86 kDa MFN1, that is consistent with diet-related inactivation. (**B**) MFN2 blots had bands at 50 kDa (mitochondrial-endoplasmic/sarcoplasmic reticulum (ER/SR) junction) and 70 kDa (OMM) [55]. Diet-exposed offspring had higher expression of 70 kDa MFN2. There was no difference in expression of the 50 kDa band. (**C**) OPA1 had two long (pro-survival) bands and three short (anti-survival) bands. Long and short bands were averaged for group comparisons. Diet-exposed males, but not females, had higher expression of short (pro-fission) OPA1. (**D**) Proposed mechanisms of impaired fusion are expressed in graphical form. (**E**) MTFP1 pro-fission and pro-mitophagy protein was more abundant in female than male hearts in every group. Diet exposure increased expression of MTFP1 in males. (**F**) Diet-exposed male, but not female hearts had higher DAP3 expression than controls. DAP3 inactivates DRP1 through Ser^637^ phosphorylation. (**G**) Downstream DRP1-Ser^637^ phosphorylation was higher in diabetes- and diet-exposed male hearts. However, diet-exposed female hearts had decreased phosphorylation that could promote fission and explain the pro-fission imbalance in female but not male diet-exposed hearts. (**H**) Proposed mechanisms of impaired fission are expressed in graphical form. Single data points represent individual offspring from different litters within each group. Data show relative expression of all bands by densitometry when compared to β-actin (mean ± SEM). Bars represent median and interquartile range. Significant differences are annotated by * for diet by two-way ANOVA or ^˄^ for interaction effects that remain by one-way ANOVA with Dunnett’s post-test; *p* ≤ 0.05. *n* = 4/group for female and male comparisons.

In our study, we identified multiple MFN1 bands: an active 70 kDa and an inactive (di-ubiquitinated) 86 kDa band [35] (Figure 5D). This is significant because mitofusin function is dependent on post-translational modifications including phosphorylation and ubiquitination [50,54]. Specifically, mono-ubiquitination (8.5 kDa) and di-ubiquitination (17 kDa) promote mitofusin degradation and impair fusion [35]. Analyzing expression of both MFN1 bands together (70 kDa + 86 kDa) uncovered a trend towards lower MFN1 in both diabetes- and high-fat diet-exposed offspring. When comparing each band separately and in both sexes, we found a sex-divergent response to prenatal exposure (Figure 6A). In females, the relative decline in cardiac MFN1 was observed for both the 70 kDa (active) and 86 kDa (inactive) bands which is consistent with overall less cardiac expression. However, in males we observed a diabetes and diet-related decline in the 70 kDa (active) band alongside an increase in the 86 kDa (inactive) band which is consistent with di-ubiquitination and inactivation of the fusion protein [35].

We also identified two MFN2 bands. One corresponded to the predicted band near 75 kDa. A second band was consistently found at 50 kDa (Figure 5B) using several anti-MFN2 antibodies (Cell Signaling 94825, Abcam AB56889, Novos H00009927-M03 and Sigma Aldrich M6310 MFN2-N terminus Ab). Garcia-Perez et al. previously characterized differences in these two bands (using Sigma Aldrich M6310) showing that the 75 kDa protein was predominantly found in fractions of isolated OMM while the 50 kDa band was predominantly found in fractions that isolated OMM, IMM, and the ER/SR together making it a marker ofmitochondria that is tethered to ER/SR [55]. Therefore, the 50 kDa band is likely a muscle specific marker of nonconventional fusion related functions including MFN2 to ER/SR communication, calcium homeostasis, network morphology, and mitophagy-induced apoptosis [56,57]. In our experiments, the total expression of MFN2 (50 kDa + 75 kDa) was significantly higher in high-fat diet-exposed offspring hearts. Analyzing the MFN2 bands separately and in both sexes, we found that this difference was primarily due to an increase in the 75 kDa band in females (Figure 6B). There was no exposure-related difference in expression of the MFN2 50 kDa band.

Post-translational processing of OPA1 from long to short bands modifies its function which includes fusion at the IMM, mitochondrial cristae structure, and resistance to apoptosis [58]. OPA1 long (pro-survival) bands are associated with fusion; short (anti-survival) bands are associated with decline in cristae density and respiration [59], as well as an increase in fission and mitophagy [58]. Measuring these separately, we found that the higher expression of OPA1 found in high-fat diet-exposed male hearts was primarily due to accumulation of short OPA1 (Figure 6C). Comparing OPA1 short:long ratio is another way to assess OPA1 processing. Control female and male hearts had a similar ratio (0.5 vs. 0.7), however, exposure to maternal high-fat diet increased the ratio 2-fold in females and 5-fold in males. This suggests that diet-exposed male hearts are prone to impaired respiration due to cristae remodeling and mitophagy.

Relative cardiac expression of DRP1 fission protein and its binding partner MFF were not affected by prenatal exposure. MFF expression is detailed in Supplemental Figure S2. However, DRP1 function is dependent on recruitment from the cytosol which is regulated by post-translational modifications, including phosphorylation (inactivation). DRP1 phosphorylation is under the influence of MTFP1 and DAP3. When both female and male offspring were analyzed together, we found no exposure-related differences in MTFP1, DAP3, or DRP1-Ser^637^ expression (Figure 6E,G). However, female controls had a 3.5-fold higher DAP3 (*p* = 0.05) and 20-fold higher DRP1-Ser^637^ phosphorylation (*p* = 0.006) than male controls. Analyzing exposure-related differences separately for female and male offspring, we found that maternal diabetes and high-fat diet altered DRP1 phosphorylation in a sex-divergent manner. Diet-exposed females had lower DRP1-Ser^637^ phosphorylation than controls, while diabetes- and diet-exposed males had higher DRP1-Ser^637^ phosphorylation than controls (Figure 6G). In males, an increase in DAP3 expression correlated downstream DRP1-Ser^637^ phosphorylation and inactivation may partially explain impaired fission in prenatally exposed male hearts. However, a diet-related increase in MTFP1 expression also occurred in males. Taken together this may lead to a diet-related increase in mitophagy in males. Female hearts had no significant difference in MTFP1 or DAP3 expression and diet-exposed hearts had lower DRP1-Ser^637^ phosphorylation favoring fission or fragmentation by non-DAP3 mediated mechanisms.

## 3. Discussion

Mitochondrial dysfunction, including impaired dynamism, contributes to the pathogenesis of adult diabetic cardiomyopathy [44,45,48,49,50,51,60]. Interestingly, there is substantial phenotypic overlap between adult diabetic cardiomyopathy and cardiac dysfunction in IDM [4,5,9,61,62]. This study demonstrated that normal developing cardiomyocytes have highly dynamic mitochondria with a relatively balanced number of fusion and fission events, but both fusion and fission were impaired by prenatal exposures. To our knowledge, we are the first to show that prenatal exposure to maternal diabetes and a high-fat diet impairs mitochondrial dynamism in the developing offspring’s heart. Since dynamism is critical for cardiac development, maturation and function, it is possible that diabetes- or diet-related impairment in mitochondrial dynamism is a key contributor to developmentally programmed heart disease in IDM which impacts cardiac health at birth and over a lifetime.

We have previously shown that maternal diabetes, especially in combination with a high-fat diet, led to impaired respiratory capacity, oxidative stress, myocardial lipid accumulation, and diastolic and systolic dysfunction in a newborn offspring’s heart [43]. In this study, we show that impaired dynamism plays a role. Our findings are summarized in Supplemental Table S4. Overall, diabetic pregnancy impaired fusion (50% decline) more than fission (28% decline) which resulted in a pro-fission imbalance, shorter, and wider mitochondria. High-fat diet during pregnancy also adversely affected dynamism causing an imbalance of fusion and fission in a sex-divergent manner. High-fat diet during diabetic pregnancy led to an equal decline in both fusion and fission that resulted in a more severe phenotype with adynamic and fragmented mitochondria. Findings demonstrate that while diabetes or diet alone can cause mitochondrial dysfunction through an imbalance of dynamism, the combination may be more detrimental, causing significantly fewer fusion and fission events that likely influence mitochondrial quality. Perhaps this is why we previously found that combination-exposed NRCM have the lowest respiratory and fatty acid oxidation capacity despite having a higher number of mitochondria (previous findings in the same model) [43]. The above findings highlight that maternal fat intake, especially during diabetic pregnancy, is a modifiable risk factor that directly affects mitochondrial dynamism and cardiac health of the developing fetus.

In addition to these important findings, we also found that in normal and exposed groups, female newborn hearts had a 2–9-fold higher expression of fusion proteins and MTFP1, but not DRP1 or MFF (Table S4). We also found a 16-fold higher expression of VDAC in female hearts compared to their male littermates. This suggests that female hearts either have a higher mitochondrial quantity or a higher fraction of healthier mitochondria. Others have reported a greater number of mitochondria in female hearts as well as shorter, wider (more fragmented mitochondria) in male hearts [63]. Additionally, they showed that females had slightly higher mitochondrial respiration, but males had more ROS production during state 4 respiration [63]. John et al. also reported a higher protein expression of mitochondrial complexes in female hearts compared to males [64]. They went on to show that in a model of type 2 diabetes, male but not female, high-fat fed New Zealand Obese (NZO) mice developed cardiac dysfunction by echocardiography [64]. In this study we found that fetal sex influenced mitochondrial dynamism contributing to gender differences in developmentally programmed heart disease. This important finding may have potential implications for the reported differences observed in the incidence and mortality rate of heart disease between men and women [65]. Additional work is necessary to clarify whether females are protected by inherent protective mechanisms that preserve healthy mitochondria.

Other studies have reported on the deleterious role of a high-fat diet on mitochondrial physiology. Chen et al. found that feeding male rats a high-fat diet for 28 weeks led to cardiomyopathy which was associated with a lower mitochondrial density/copy number, greater variation in size, and abnormal cristae structure consistent with impaired dynamism, biogenesis, and quality control [66]. However, they did not report the effect of high-fat feeding on female hearts. Although not cardiac specific, Khamoui et al. also examined the effects of pre- and post-natal high-fat diet on mitochondrial function in skeletal muscle [67]. Like us, they found a sexually divergent response to prenatal high-fat diet; diet-exposed females who remained on a high-fat diet into adulthood (compared to post-weaning transition to normal chow) had impaired respiratory capacity of skeletal muscle, but males had increased respiratory capacity if they maintained a high-fat diet into adulthood [67]. Another recent study by Ostadal et al. adds evidence that gender differences in mitochondria bestow cardioprotection to females following ischemia and reperfusion injury well into adulthood [68]. In our study we showed that a high-fat diet during pregnancy impairs dynamism in a sex-divergent manner. Both male and female NRCM had approximately 45% decline in fission, but the decline in fusion was dependent on fetal sex. Females had a 65% decline in fusion resulting in a pro-fission balance, while males had only a 24% decline in fusion with an anti-fission balance that could adversely affect replication/biogenesis and mitochondrial quality control mechanisms. Taken together, our study adds growing support that prenatal exposures to high-fat diet can impair mitochondrial dynamism in a gender-specific manner to influence cardiac development, bioenergetics, and aging which directly contributes to gender discrepancies in developmentally programmed disease.

This study goes a step further to identify fusion and fission proteins as targetable, pathogenic regulators of heart health in the fetus exposed to excess circulating maternal fuels. Although we found little difference in the expression of common genes regulating dynamism in the heart, protein expression and post-translational modifications were identified. This suggests that excess circulating maternal fuels or associated metabolic changes in the maternal–placental–fetal environment (inflammation, ROS, hormonal) can incite direct, adaptive mitochondrial responses in the developing fetus. Specifically, MFN1 expression was approximately 50% lower in diabetes, diet, and combination exposed offspring hearts. We speculate that this is the primary cause of impaired fusion found in all exposure groups. The sex-divergent decline in fusion following diet exposure supports this. Diet-exposed females had a 65% decline in fusion events, but males had only a 25% decline. This may be because normal female hearts had a higher MFN1 expression than male hearts, so exposure led to a more robust decline in relative expression and function. Moreover, diet-exposed females had a lower expression of all MFN1 bands (active and inactive) compared to controls, but diet-exposed males had a lower expression of the 70 kDa (active) MFN1 band alongside an increase in the 86 kDa (inactive) MFN1, which is more consistent with di-ubiquitination (inactivation) which marks mitochondria for mitophagy [35]. We also speculate that this sex-divergent response contributes to poorer mitochondrial quality in exposed male hearts.

We also found that prenatal exposure to a high-fat diet led to an increase in both the *Mfn2* gene in males and MFN2 protein expression in both females and males. When only fusion-related effects are considered, an increase in MFN2 may seem beneficial. However, MFN2 has many non-fusion related roles including ER/SR communication, calcium homeostasis, mitochondrial reticulum formation, and regulation of mitophagy induced apoptosis [55,56,57,69]. Specifically, in the heart, MFN2 predisposes cardiomyocytes to mitochondrial permeability transition and cell death following stress or ischemia/reperfusion [69,70]. Over expression of *Mfn2* has reportedly been associated with impaired cell proliferation through the Ras-Raf-ERK signaling pathway [71]. This points to a feasible pathogenic mechanism of cardiomyopathy in IDM because higher expression of MFN2 during cardiac development could contribute to a decreased number of cardiomyocytes, secondary hypertrophy, and impaired diastolic and systolic function.

Interestingly, MFN1 and MFN2 expression influence OPA1 activity at the IMM. Specifically, OPA1 requires MFN1 to promote fusion. Conversely, OPA1 requires MFN2 for proteolytic cleavage to short forms that decrease cristae density and subsequent respiratory capacity [59,72,73]. In our study, both MFN2 and OPA1 protein expression were higher in diet-exposed male hearts; and the increase in OPA1 was primarily due to an increase in short bands, consistent with OPA1 cleavage. Tsushima et al. have also shown that myocardial lipotoxicity induces OPA1 cleavage to promote fragmentation, ROS generation, and mitochondrial reticulum remodeling [36]. Their work supports our findings that OPA1 processing is one mechanism involved in myocardial lipotoxicity. Our work goes one step further to show that second-hand exposure to excess circulating maternal lipids can impair fusion, respiratory capacity, and ultimately ventricular function in high-fat diet-exposed newborn offspring [43].

Prenatal exposures also influenced expression and post-translational modification of fission-related proteins in a sex-divergent manner. Diet-exposed males had a higher expression of MTFP1 which recruits DRP1 to the mitochondrial membrane. MTFP1 is a downstream target of the PI3K-AKT pathway which is under the influence of insulin signaling [38,74]. This is of interest to us because we have previously observed fetal hyperinsulinemia and downstream alterations of PI3K-AKT activity in our model [53]. We propose that in our study, MTFP1 incites mitochondrial fragmentation by non-fission mediated mechanisms. This theory stems from the fact that diet- and combination-exposed male NRCM had 42% fewer fission events than controls even though they had higher MTFP1 expression. This is likely due to an opposing diet mediated DRP1-Ser^637^ phosphorylation (inactivation) that was also found in male hearts. Indeed, Zhang et al. showed that Drp1-Ser^637^ must be dephosphorylated in order for it to interact with MFF [75]. Unlike MFF and MiD49/51 which are competitive DRP1 binding partners on the OMM [76], MTFP1 is located on the IMM. It is feasible that simultaneous DAP3-mediated phosphorylation (inactivation) of DRP1-Ser^637^ alongside increased MTFP1 leads to fragmentation (leaking IMM) that incites mitophagy-associated cell death. DAP3 is a mitochondrial ribosomal protein that localizes to the mitochondrial matrix. Work by Xiao et al. showed that DAP3 regulates phosphorylation (inactivation) of DRP1-Ser^637^ and retention time of DRP1 on the OMM which sensitizes cells to mitochondrial-mediated cell death [40]. If proven, this may be another mechanism contributing to poorer mitochondrial quality in male NRCM which sets the foundation for gender differences in developmentally programmed heart disease. Indeed, diet-exposed females in our study did not have higher expression MTFP1 or DAP3. Conversely, they had a relative de-phosphorylation of DRP1-Ser^637^ which would favor fission and fragmentation. These sex-specific differences in DRP1 phosphorylation partially explain why females exposed to maternal high-fat diet (but not diabetes) had a pro-fission imbalance (1.7 fission:fusion ratio) and males did not (0.88).

More recent studies have demonstrated that dynamic changes regulate mitochondrial function by variable oligomerization and site-specific phosphorylation, ubiquitination, summolation, and O-GlcNAcylation [77]. For example, Pyakurel et al. showed that MFN1 function is altered by ERK-mediated phosphorylation at threonine 562 which resulted in fragmentation, BAK oligomerization, and cell death by cytochrome c release [54]. Insulin and insulin growth factor, which are elevated in developing offspring exposed to diabetic pregnancy, are common regulators of ERK activity. Furthermore, Toyama et al. showed that AMPK-regulated phosphorylation of MFF at Ser155 and Ser172 regulates fission and mitophagy in response to cellular metabolic signals [78,79]. Additional studies are needed to test these post-translational modifications as well as targetable upstream regulators of dynamism including MAPK, ERK, and AMPK [80] with the goal to improve mitochondrial and cardiovascular health in IDM.

## 4. Methods

### 4.1. Animal Model

Animal care followed guidelines set forth by the Animal Welfare Act, The National Institutes of Health Guide for the Care and Use of Laboratory Animals under approval from the Sanford Institutional Animal Care and Use Committee (Protocol 93-08017B, initially approved 8/8/2015). As previously described [42,43,52,53,81], female Sprague–Dawley rats (Harlan Laboratories Inc., Indianapolis, IN, USA) received either a control diet with 18% of daily calories as fat (TD2018 Teklad, Harlan Laboratories, Madison, WI, USA) or a high-fat diet with 40% of calories as fat (TD95217 custom diet Teklad, Harlan Laboratories, Madison, WI, USA) for 28 days prior to timed-breeding with males. On gestational day 14 either citrate buffer (diluent) or streptozotocin (65 mg/kg) was administered for diabetes induction. Twice daily blood glucose levels were measured by tail nick sampling and insulin was administered to keep glucose in a target range of 200–400 mg/dL. Dams delivered spontaneously on gestational day 22 yielding newborn offspring from four groups: controls, diabetes-exposed, high-fat diet-exposed, and combination-exposed. Newborn offspring from each litter were euthanized by cervical dislocation. Hearts were harvested for primary cardiomyocyte isolation or snap frozen and stored at –80 °C for RNA and protein. Maternal and offspring characteristics, including echocardiography, have been well-described elsewhere [43,52,53,81]. In short, dams fed a high-fat diet gained more weight. Diabetic rats had hyperglycemia in the last third of pregnancy. Diabetic and high-fat-fed dams developed higher triglyceride levels and combination-exposed dams developed marked hypertriglyceridemia (>5-fold higher). Offspring exposed to diabetes had significantly higher insulin levels which was exacerbated by combination exposure. High-fat diet-exposed litters had more than 4-fold higher perinatal mortality rate [52]. Pups that survived developed diastolic and systolic dysfunction, with combination-exposed offspring being most severely affected [43].

### 4.2. Isolation of Neonatal Ventricular Rat Cardiomyocytes

Newborn offspring hearts were harvested to Hank’s Balanced Salt Solution (HBSS) on ice. The aorta was flushed with PBS and the atria was removed. Neonatal ventricular rat cardiomyocytes (NRCM) were isolated using a 0.15% trypsin and DNase digestion, as previously described [42,43,81]. After pelleting, cells were re-suspended in Dulbecco’s Modified Eagle Medium-1 (DMEM supplemented with 10% bovine serum albumin and 1% penicillin/streptomycin), then plated to an uncoated 35 mm dish and incubated for 1 h (5% CO_2_, 37 °C) so that rapidly attaching fibroblasts could be separated. NRCM were lifted and transferred to 0.1% gelatin coated plates in DMEM-2 (DMEM-1 + 100 μM bromodeoxyuridine or BrdU) for incubation (5% CO_2_, 37 °C) until used for confocal live-cell imaging within hours after isolation and plating.

### 4.3. Confocal Live-Cell Imaging and Quantification of Mitochondrial Dynamism

Isolated NRCM were pooled from 2–4 female or 2–4 male same-sex pups per litter. NRCM (150,000 cells) were plated in DMEM-2 to a 35 mm glass bottom FluoroDish (FD3510, World Precision Instruments). NRCM were stained with 2 µM Hoechst (AS-83218, AnaSpec Inc.) for nuclei, 1 µM MitoTracker Green FM (M7514, Thermo Fisher Scientific) for mitochondrial identification, and 20 nM tetramethylrhodamine ethyl ester (TMRE) Red (T669, Thermo Fisher Scientific) to assess the mitochondrial membrane potential [43]. Three to ten cells/plate were imaged (60×) at baseline and a rate of 6 s per acquisition (no delay) for 5 min using a Nikon Eclipse A1 confocal laser microscope system with a stage top incubator (Supplemental Video S1). Images were analyzed in blinded fashion using NIS-Elements Software with a 10 µm grid overlay and measuring tool. Mitochondrial length and width were recorded by systematically measuring 10 mitochondria/cell in at least two representative cells/sample, starting around the nucleus and working out peripherally through the grid overlay. The average of 20 mitochondria/sample was used for comparisons. Dynamism was evaluated by counting the number of fusion and fission events observed in each 100 µm^2^ square grid. A fission event was recorded when one strand of stained mitochondria divided in two or more smaller strands of mitochondria. Care was taken that no mitochondria smaller than 2 µm was used, because this indicates mitochondrial fragmentation. Fusion was noted when two separate mitochondria combined and formed one longer mitochondrion. Note that this method is limited by the ability to count events imaged in one plane. Dynamism was calculated as the average number of events/cell/5 min video, as well as the events/100 µm^2^/5 min video to account for potential variance in cell size. A fission:fusion ratio was calculated to detect imbalanced dynamism.

### 4.4. Quantitative Real-Time PCR

Newborn (P1) rat whole hearts from different litters were homogenized in RLT buffer using Precellys 24 lysis and homogenization system (Bertin Technologies, Rockville, MD). RNA was extracted using the RNeasy Fibrous Tissue Mini kit (Qiagen, Germantown, MD, USA). RNA integrity was assessed by electropherograms using 2100 BioAnalyzer (Agilent Technologies, Santa Clara, CA, USA) and demonstrated RNA Integrity Numbers of 9.2–10 (average = 9.8). RNA concentration was measured by Epoch spectrophotometer (BioTek, Winooski, VT). Using 1 ug of RNA, complementary DNA (cDNA) was synthesized using iScript cDNA Synthesis Kit and T100 Thermal Cycler (Bio-Rad, Hercules, CA, USA) via manufacturer’s protocol. Quantitative PCR (qPCR) was performed by TaqMan approach in an ABI7500 qPCR system with Absolute Blue qPCR Mix (ThermoFisher, Waltham, MA, USA). Beta-2-microglobulin (*B2m*) was used as the reference gene. Probe/primer sets were obtained from ThermoFisher (Waltham, MA, USA) and Integrated DNA Technologies (Coralville, IA, USA) and are detailed in Supplemental Table S1.

### 4.5. Protein Analyses

Newborn (P1) rat whole hearts from different litters were homogenized and sonicated in radioimmunoprecipitation assay buffer (50 mM Tris (pH 7.5), 150 mM NaCl, 1% Triton X, 0.5% deoxycholate, 0.1% sodium dodecyl sulfate) with cOmplete™ protease inhibitor cocktail (Roche, Indianapolis, IN, USA) and phosphatase inhibitor cocktail (Sigma-Aldrich, St. Louis, MO, USA). Protein concentrations were measured using the DC Protein Assay kit (Bio-Rad, Hercules, and CA, USA) and a SpectraMax M5 (Molecular Devices, Sunnyvale, CA, USA). Samples were prepared using Laemmli buffer and reducing agent then subjected to electrophoresis on 4–15% Criterion TGX™ Gels using Tris/Glycine/SDS buffer (Bio-Rad). MagicMark™ XP Western Protein Standard and EZ-RUN™ Pre-stained Recombinant Protein ladder 10-170 KDa (ThermoFisher, Waltham, MA, USA) were used to identify band size. Gels were transferred to polyvinylidene difluoride membranes using Trans-Blot Turbo™ Transfer System (Bio-Rad). Membranes were dried, rehydrated in methanol, washed 2 times in TBS for 2 min each, blocked in TBS containing 10% Clear Milk Blocking Buffer (ThermoFisher) for 10 min and then incubated overnight at 4 °C with primary antibody as specified in Supplemental Table S2. After washing 2 times in TBS-T for 2 min each, membranes were blocked again and incubated with secondary antibody of goat anti-rabbit IgG-HRP (Southern Biotech, Birmingham, AL, USA) or donkey anti-mouse IgG IRDye 800CW (LI-COR, Lincoln, NE, USA) for 1 h. Bands were visualized using Luminata Forte HRP Chemiluminescence Substrate (ThermoFisher). Images were captured using a LI-COR Oydssey Imaging System and analyzed using VisionWorks LS Software (UVP, Upland, CA, USA). Reference protein was β-actin. Voltage-dependent anion channel or porin (VDAC), an OMM protein, was used as a secondary reference and a VDAC:actin ratio was calculated as a way to identify differences in mitochondria number/sample.

### 4.6. Statistics

Statistical analyses were performed using GraphPad Prism 5 software (La Jolla, CA, USA). Group data were averaged and expressed as mean ± SEM. Diabetes, diet, and interaction effects were interrogated using two-way ANOVA with Bonferroni post hoc test. When a significant interaction was found, two-way ANOVA was invalid and so differences between controls and each exposed group were interrogated using one-way ANOVA and Dunnett’s post hoc test. Sex-specific differences were evaluated using two-tailed, unpaired, Student’s *t*-test. Differences were considered statistically significant at a value of *p* < 0.05.

## 5. Conclusions

In conclusion, this study identifies mitochondrial dynamism and potential upstream regulators as targetable mechanisms involved in the pathogenesis of cardiomyopathy in infants born to diabetic or obese mothers. The value of this study comes in four key points. First, we are one of the first to demonstrate that exposing the developing fetal heart to excess circulating maternal fuels including glucose, lipid, or both, impairs both fusion and fission during critical periods of development which could impact heart health over a lifetime. Second, we compared four offspring groups to show that either maternal diabetes or a high-fat diet alone can impair mitochondrial dynamism by overlapping mechanisms. Importantly, a high-fat diet in combination with diabetic pregnancy exacerbates mitochondrial dysfunction and serves as a modifiable risk factor that may improve outcomes. Third, we showed that an offspring’s gender influences mitochondrial dynamism and may confer cardioprotective effects in female hearts. Fourth, we uncovered potential mechanisms of mitochondrial dysfunction that serve as targetable pathways for intervention. Our findings add to a growing foundation of knowledge needed to develop pre- and post-natal interventions that will prevent developmentally programmed disease in IDM.

## Figures and Tables

**Table 1:** An offspring’s sex influences mitochondrial dynamism and morphology in neonatal rat cardiomyocytes.

Event	Offspring Group	Female Mean (SEM)	Male Mean (SEM)	*p*-Value
**Fusion**	Controls	1.03 (0.12)	0.82 (0.14)	0.31
**(events/100 μm^2^/5 min)**	Diabetes exposed	0.53 (0.13)	0.39 (0.06)	0.42
	Diet exposed	**0.35 (0.05)**	**0.58 (0.08)**	0.04 *
	Combination exposed	0.58 (0.10)	0.42 (0.06)	0.18
**Fission**	Controls	0.98 (0.16)	0.84 (0.11)	0.46
**(events/100 μm^2^/5 min)**	Diabetes exposed	0.66 (0.06)	0.64 (0.05)	0.72
	Diet exposed	0.52 (0.03)	0.48 (0.05)	0.47
	Combination exposed	0.42 (0.13)	0.49 (0.02)	0.55
**Fission:Fusion**	Controls	0.91 (0.09)	1.14 (0.17)	0.40
**(Ratio)**	Diabetes exposed	1.87 (0.48)	1.80 (0.30)	0.68
	Diet exposed	1.71 (0.13)	0.88 (0.16)	0.06
	Combination exposed	**0.77 (0.19)**	**1.27 (0.05)**	0.02 *
**Length**	Controls	5.26 (0.26)	5.08 (0.36)	0.73
**(μm)**	Diabetes exposed	4.17 (0.20)	4.36 (0.30)	0.58
	Diet exposed	4.53 (0.32)	4.41 (0.23)	0.79
	Combination exposed	3.88 (0.26)	4.15 (0.22)	0.44
**Width**	Controls	**0.59 (0.03)**	**0.73 (0.03)**	0.008 *
**(μm)**	Diabetes exposed	0.76 (0.03)	0.85 (0.03)	0.06
	Diet exposed	0.83 (0.03)	0.88 (0.04)	0.37
	Combination exposed	0.77 (0.05)	0.77 (0.05)	0.41

NRCM, neonatal rat cardiomyocytes. Significant differences (*p* ≤ 0.05) between female and male offspring by *t*-test are indicated by boldface and *.

**Table 2:** Sex-specific differences in expression of proteins involved in mitochondrial dynamism.

Protein	Group	Female Mean (SEM)	Male Mean (SEM)	*p*-Value
**MFN1**	Controls	21.95 (11.39)	2.40 (1.01)	0.14
	Diabetes exposed	**6.10 (0.73)**	**2.24 (0.65)**	0.007 *
	Diet exposed	**7.12 (0.73)**	**2.15 (0.25)**	<0.001 *
	Combination exposed	**10.08 (1.15)**	**1.30 (0.19)**	<0.001 *
**MFN2**	Controls	**0.80 (0.09)**	**0.09 (0.07)**	<0.001 *
	Diabetes exposed	**0.76 (0.10)**	**0.31 (0.10)**	0.02 *
	Diet exposed	**1.04 (0.19)**	**0.47 (0.15)**	0.05 *
	Combination exposed	**1.11 (0.07)**	**0.59 (0.07)**	0.001 *
**OPA1**	Controls	1.73 (0.55)	0.60 (0.30)	0.12
	Diabetes exposed	**1.39 (0.32)**	**0.49 (0.17)**	0.05 *
	Diet exposed	**1.76 (0.31)**	**0.90 (0.16)**	0.05 *
	Combination exposed	1.85 (0.64)	1.94 (0.61)	0.93
**DRP1**	Controls	2.43 (0.29)	2.63 (0.67)	0.79
	Diabetes exposed	2.09 (0.27)	1.99 (0.39)	0.83
	Diet exposed	2.03 (0.31)	1.95 (0.32)	0.86
	Combination exposed	**3.03 (0.27)**	**1.39 (0.12)**	0.001 *
**MFF**	Controls	1.91 (0.52)	1.70 (0.29)	0.73
	Diabetes exposed	1.65 (0.29)	1.42 (0.26)	0.58
	Diet exposed	2.37 (0.62)	1.46 (0.20)	0.21
	Combination exposed	1.50 (0.81)	2.20 (0.32)	0.45
**MTFP1**	Controls	**1.66 (0.49)**	**0.14 (0.17)**	0.02 *
	Diabetes exposed	**2.27 (0.25)**	**0.17 (0.09)**	<0.001 *
	Diet exposed	**2.17 (0.30)**	**0.33 (0.07)**	<0.001 *
	Combination exposed	3.41 (1.54)	0.42 (0.12)	0.10
**VDAC**	Controls	**7.20 (0.99)**	**0.44 (0.06)**	<0.001 *
	Diabetes exposed	**6.24 (0.78)**	**0.56 (0.06)**	<0.001 *
	Diet exposed	**5.90 (0.51)**	**0.51 (0.05)**	<0.001 *
	Combination exposed	**8.65 (1.68)**	**0.46 (0.05)**	0.003 *

Whole heart protein lysate from newborn pups from separate litters were used to assess levels of mitochondrial dynamism regulating proteins by Western blot. Values are expressed as a mean ± SEM expression relative to beta-actin, the reference protein. Significant sex-specific differences by *t*-test (*p* ≤ 0.05) are boldface and indicated with *. *n* = 4 males and 4 females/group.

## Supplementary Materials

Supplementary materials can be found at https://www.mdpi.com/1422-0067/20/12/3090/s1.
