# Supplementary material for: Diabetic Pregnancy and Maternal High-Fat Diet Impair Mitochondrial Dynamism in the Developing Fetal Rat Heart by Sex-Specific Mechanisms
Source: Int J Mol Sci. 2019 Jun 25;20(12):3090. doi: 10.3390/ijms20123090 (PMC6627740; doi:10.3390/ijms20123090)
Supplement: Supplementary file 1 [file ijms-20-03090-s001.zip › Supplementary.pdf]

Table S1. Primers and their target sequence

Gene	Manufacturer	Ref Seq	Assay ID	Sequence
Mfn1	Integrated DNA Technologies	NM_138976	Rn.PT.58.44207597	Probe: 5'-/56-FAM/cagcgttga/ZEN/ttccgagtttgcca/3IABkFQ/-3'<br>P1: 5'-ccgctcattcaccttatggaa-3'<br>P2: 5'-gattgataagttctgccttgatgc-3'
Mfn2	Integrated DNA Technologies	NM_130894	Rn.PT.58.13375660	Probe: 5'-/56-FAM/ccagctaga/ZEN/aacgagatgtccctgc/3IABkFQ/-3'<br>P1: 5'-ccatgtgtcgcttatccttct-3'<br>P2: 5'-tgactccagccatgtccat-3'
Opa1	Integrated DNA Technologies	NM_133585	Rn.PT.58.6858853	Probe: 5'-/56-FAM/tcacatgcg/ZEN/ttgacactcgttccc/3IABkFQ/-3'<br>P1: 5'-tgttcctgagttcatggtctg-3'<br>P2: 5'-ctgagccaggttactccaaag-3'
Drp1 (Dnm1l)	Integrated DNA Technologies	NM_053655	Rn.PT.58.35587248	Probe: 5'-/56-FAM/cagtacccg/ZEN/catccatgagatcaagc/3IABKFQ/-3'<br>P1: 5'-aacccttcccatcaatacatcc-3'<br>P2: 5'-tccagagaggtagatccagatg-3'
Mff	Integrated DNA Technologies	NM_001039015	Rn.PT.58.46192959.g	Probe: 5'-/56-FAM/tgccagtgt/ZEN/gataatgcaagtccca/3IABkFQ/-3'<br>P1: 5'-ttatttcctgtaaccacgatcct-3'<br>P2: 5'-caaatgctgacctggaacaag-3'
B2m	ThermoFisher	NM_012512.2	Rn00560865_m1	Amplicon:<br>CTGCTGACCGGACCGGCACGATGGCTCGCTCGGTGACCGTGATCTTTCTGGTGCTTGTCTCTCTGGCCGT<br>CGTGCTTGCCATTCAGAAAACTCCCCAAATTCAAGTGTACTCTCGCCATCCACCGGAGAATGGGAAGCCC<br>AACTTCCTCAACTGCTACGTGTCTCAGTTCCACCCACCTCAAATAGAAATTGAGCTACTGAAGAATGGAA<br>AGAAGATACCAAATATCGAGATGTCA

Table S2. Antibodies used for Western Blot analyses.

Antibody	Expected Size	Protein Loaded	Antibody Dilution	Manufacturer	Catalog #	Host Species	Secondary Reporter
MFN1	70, 86kDa	20ug	1:200	Santa Cruz	SC50330	Rabbit[ǂ]	HRP 1:5000
MFN2	50, 70kDa	20ug	1:1000	Sigma	M6319	Rabbit	HRP 1:5000
OPA1	80, 100kDa	20ug	1:1000	Cell Signaling	80471s	Rabbit	HRP 1:5000
DRP1	84kDa	20ug	1:350	Santa Cruz	SC-32898	Rabbit[ǂ]	HRP 1:5000
MFF	27,33,38kDa	20ug	1:1000	Cell Signaling	84580S	Rabbit	HRP 1:5000
MTFP1	14kDa	20ug	1:1000	Antibodies-Online	ABIN3047683	Rabbit	HRP 1:5000
DAP3	35kDa	20ug	1:1000	Becton Dickinson	610662	Mouse	IRDye 1:5000
p-DRP1	100, 80kDa	20ug	1:500	Cell Signaling	4867S	Rabbit	HRP 1:5000
Actin	45kDa	20ug	1:1000	Cell Signaling	5125S	Rabbit	HRP 1:5000
VDAC	39kDa	20ug	1:1000	Abcam	AB14734	Mouse	IRDye 1:5000

[ǂ] Discontinued. HRP, goat anti-rabbit IgG-HRP (Southern Biotech, Birmingham, AL); IRDye (LI-COR, Lincoln, NE), donkey anti-mouse IgG IRDye 800CW.

Table S3. Female and male differences in expression of genes involved in mitochondrial dynamism.

**Gene**	**Offspring Group**	**Female**<br>Mean (SEM)	**Male**<br>Mean (SEM)	**P value**
Mfn1	Controls	0.82 (0.12)	0.48 (0.21)	0.16
	Diabetes exposed	0.60 (0.09)	0.52 (0.07	0.39
	Diet exposed	0.69 (0.07)	0.63 (0.06)	0.58
	Combination exposed	0.69 (0.08)	0.65 (0.07)	0.14
Mfn2	Controls	0.16 (0.02)	0.10 (0.02)	0.15
	Diabetes exposed	0.13 (0.02)	0.12 (0.01)	0.68
	Diet exposed	0.15 (0.03)	0.15 (0.01)	0.98
	Combination exposed	0.13 (0.02)	0.16 (0.02)	0.14
Opa1	Controls	0.18 (0.02)	0.11 (0.02)	0.15
	Diabetes exposed	0.20 (0.03)	0.15 (0.01)	0.15
	Diet exposed	0.18 (0.03)	0.18 (0.02)	0.79
	Combination exposed	0.19 (0.01)	0.16 (0.02)	0.46
Drp1	Controls	0.12 (0.02)	0.07 (0.01)	0.07
	Diabetes exposed	0.11 (0.02)	0.09 (0.01)	0.26
	Diet exposed	0.12 (0.02)	0.11 (0.01)	0.69
	Combination exposed	**0.14 (0.02)**	**0.09 (0.01)**	**0.04***
Mff	Controls	0.14 (0.02)	0.11 (0.03)	0.46
	Diabetes exposed	0.13 (0.03)	0.10 (0.01)	0.37
	Diet exposed	0.13 (0.02)	0.16 (0.04)	0.46
	Combination exposed	0.14 (0.02)	0.10 (0.01)	0.07

Whole heart mRNA levels of mitochondrial dynamism regulating genes were determined by qPCR. Values are expressed as a mean ± SEM expression relative to beta-2-microglobulin (*B2m*), the reference gene. Significant sex-specific differences are bolded and indicated with * by t-test ($p \leq 0.05$). N=8-9 males and 8 females/group.

Table S4. Summary of prenatal exposure and gender related differences in cardiac mitochondrial dynamism.

		Female	Male	Gender Differences
Morphology	Fusion Events	↓ in all groups	↓ in all groups	Diet impairs ♀ fusion more than ♂
	Fission Events	↓ in all groups	↓ in diet exposed	None, both impaired
	Length	↓ with diabetes	Trend ↓ in all	None, similar trend
	Width	↑ in all groups	Trend ↑ in all	Control ♂ wider than ♀
Genes	Fusion Genes	No difference	MFN2 ↑ in diet exposed	None, similar trend
	Fission Genes	No difference	No difference	Combination exposed ♀>DRP1 than ♂
Proteins	Fusion Proteins			
	MFN1	Trend ↓ in overall expression	↑ ubiquitination (inactivation of MFN1) in diet exposed	All ♀ > expression than ♂
	MFN2	↑ in diet exposed	↑ in diet exposed	All ♀ > expression than ♂
	OPA1	No difference	↑ long to short ratio (inactivation of OPA1) in diet exposed	♀ > expression than ♂ with no evidence of inactivation
	Fission Proteins			
	DRP1	↓ Ser^{637} phosphorylation to activate fission in diet exposed	↑ Ser^{637} phosphorylation to impair fission in all exposed	♀ > expression than ♂ in combination exposed. Sex-divergent phosphorylation via DAP3 (favors mitophagy males)
	MTFP1	No difference	↑ in diet exposed	♀ > expression than ♂ except in combination exposed
	DAP3	No difference	↑ in diet exposed	♀ > expression than ♂ except in combination exposed
	VDAC	No difference	No difference	All ♀ > expression than ♂

Group differences are designated by ↑ for higher or ↓ for lower. ♀, female offspring hearts; ♂, male offspring hearts.

Supplemental Figure S1. Fusion and fission events per cell and per area to account for cell size.

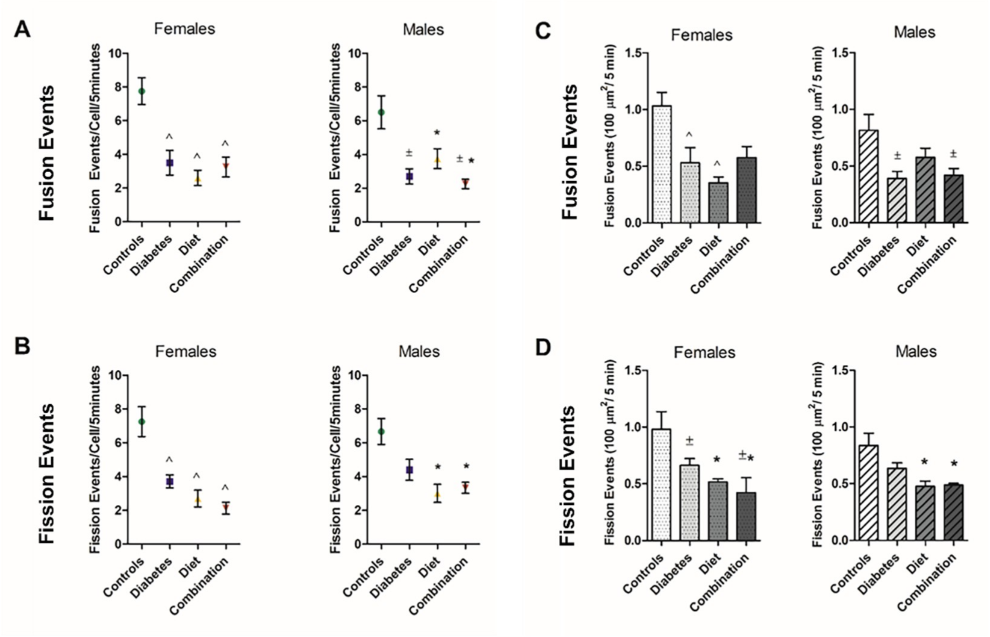

Figure S1. Maternal diabetes and high fat diet impair mitochondrial dynamism in newborn rat cardiomyocytes. Using confocal live cell imaging, we compared the average number of (A) fusion and (B) fission events/cell in a 5 minute recording of primary neonatal cardiomyocytes from controls and those exposed to maternal diabetes, high-fat diet, or the combination. To account for variability in cell size/image, the number of (C) fusion and (D) fission events/100µm2 (10 x 10 µm square) in a 5 minute recording were also compared; the outcome remained similar. Data in A-B is the median +/- interquartile range and is the same data presented in Figure 2, but repeated here for ease of comparison between events/time and events/area/time. Data in C-D is mean +/- SEM. N=4-7/group for females and n=4-6/group for males. Significant differences ($p \leq 0.05$) are indicated with ± for diabetes effect, * for dietary effect by two-way ANOVA and ^ for interaction with significance remaining by one-way ANOVA with Dunnett's post hoc analysis.

Supplemental Figure S2. Mitochondrial fission factor expression in exposed offspring hearts.

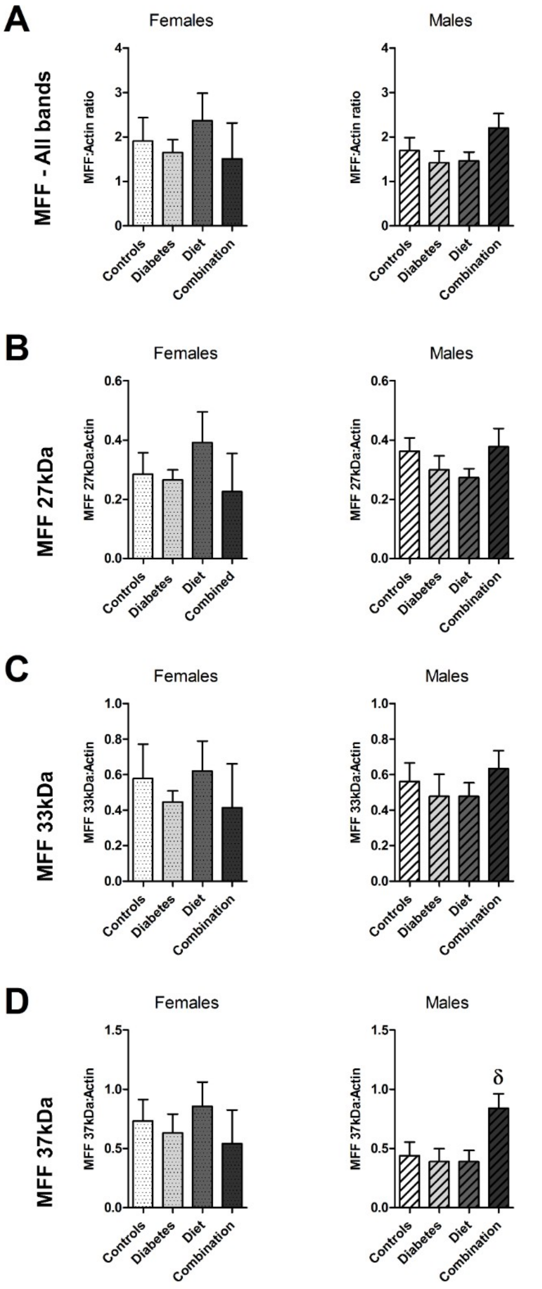

Figure S2. Mitochondrial fission factor (MFF) expression in newborn rat hearts. Whole heart protein lysate from control, diabetes, high-fat diet, and combination exposed newborn rats was analyzed for difference in expression of mitochondrial fission factor (MFF). MFF blots revealed three bands at 27, 33 and 37 kDa. These different size proteins likely represent known post-translational modifications known to affect function including AMPK regulated phosphorylation of MFF at Ser155 and Ser172 which regulates fission and mitophagy in response to cellular metabolic signals. While combination exposed males had a higher 37kDa MFF than females, there was no significant exposure related difference found. Data is shown for all offspring for females (n=4/group) and males (n=4/group) separately. The significant gender-related difference ($p \le 0.05$) is indicated with $^{\delta}$ by T-test.
